# Hydrothermal Synthesis of Polyhedral Nickel Sulfide by Dual Sulfur Source for Highly-Efficient Hydrogen Evolution Catalysis

**DOI:** 10.3390/nano10112115

**Published:** 2020-10-24

**Authors:** Yuan Gao, Ka Wang, Zixia Lin, Haizeng Song, Xiaomeng Duan, Zehui Peng, Shancheng Yan

**Affiliations:** 1 School of Geography and Biological Information, Nanjing University of Posts and Telecommunications, Nanjing 210023, China; 1218063835@njupt.edu.cn (Y.G.); 1217063729@njupt.edu.cn (K.W.); 1019172222@njupt.edu.cn (Z.P.); 2 Testing Center, Yangzhou University, Yangzhou 225009, China; zxlin@yzu.edu.cn; 3 School of Electronic Science and Engineering, Nanjing University, Nanjing 210093, China; dg1823028@smail.nju.edu.cn; 4 Jiangsu National Synergetic Innovation Center for Advanced Materials (SICAM), Institute of Advanced Materials (IAM), Nanjing University of Posts & Telecommunications, Nanjing 210023, China; 1218063822@njupt.edu.cn

**Keywords:** transition metal sulfide, hydrogen evolution reaction, electrocatalyst

## Abstract

Transition metal sulfides are cheap and efficient catalysts for water splitting to produce hydrogen; these compounds have attracted wide attention. Nickel sulfide (NiS_2_) has been studied in depth because of its simple preparation process, excellent performance and good stability. Here, we propose a modification to the hydrothermal synthesis method for the fabrication of a highly efficient and stable NiS_2_ electrocatalyst prepared by two different sulfur sources, i.e., sulfur powder and C_3_H_7_NaO_3_S_2_ (MPS), for application in hydrogen evolution reactions. The obtained NiS_2_ demonstrated excellent HER performance with an overpotential of 131 mV to drive -10 mA cm^−1^ in 0.5 M H_2_SO_4_ solution with 5mV performance change after 1000 cycles of stability testing. We believe that this discovery will promote the industrial development of nonprecious metal catalysts.

## 1. Introduction

Fossil fuels have been extremely important over the past hundred years, at the same time causing very serious damage to the environment [1,2,3]. In order to alleviate the pressure on the ecological environment, people have begun to look for more green, efficient and low-cost methods of energy generation [4,5,6]. Hydrogen is a clean energy source; it has a calorific value higher than fossil fuels and may be produced from water without pollution to the environment [7]. Therefore, the usage of catalysts to optimize the decomposition of water for hydrogen production has attracted great attention from researchers [8,9,10,11,12]. At the present, there are a variety of extremely stable and high-efficiency catalysts, such as platinum and palladium; however, their application is limited by their high cost [13,14]. Thus, there is a need to find a low-cost and stable catalyst.

Nonprecious transition metals are abundant and exhibit a good performance for hydrogen evolution reactions [15]. Nonprecious transition metal derivatives such as oxides, sulfides, carbides, nitrides, and alloys have been studied, and all demonstrated good catalytic performance. As a transition metal, nickel-based materials have been thoroughly studied in terms of catalysis [1,16,17,18,19]. Nickel-based dichalcogenides, specifically, NiS_2_, are narrow-bandwidth semiconductors performing well in acidic environments with a relatively small Tafel slope and a low overpotential [17,20,21,22,23]. Some NiS_2_ crystals have irregular morphologies and complicated structures, causing instability in the performance of the catalyst. If there is further improvement in the catalytic performance and stability of NiS_2_, it is expected to be widely used in industry.

In this work, we propose a hydrothermal synthesis method of polyhedral NiS_2_ (MS) from C_3_H_7_O_3_NaS_2_ (MPS) and sulfur powder used as the dual sulfur source. The obtained NiS_2_ was used for hydrogen evolution reactions (HER), performed in an acidic environment of 0.5M H_2_SO_4_. The catalyst showed excellent performance: the turn-on voltage at a current density of 10 mA cm^−2^ was 131 mV, and the Tafel slope was 50 mV dec^−1^. In order to ensure the relevance of this technique for practical applications, we also conducted a stability test of 1000 circles; the resulting performance change was only 5 mV. For the renewable energy industry, we believe that this method of preparation of NiS_2_ electrodes will promote the development of electrocatalysts.

## 2. Materials and Methods 

### 2.1. Materials and Chemicals 

The carbon fiber paper (CFP) model was obtained from CeTech Co., Ltd. (WOS1009) (Taichung City, Taiwan). Ni(NO_3_)_2_·6H_2_O was obtained from Shanghai Titan Technology Co., Ltd. (Shanghai, China). Sulfur powder (S) was obtained from Nanjing Chemical Reagent Co., Ltd. (Nanjing, China). Sodium 3-mercapto-1-propanesulfonate (C_3_H_7_NaO_3_S_2_, Na[SH(CH_2_)_3_SO_3_], MPS) was obtained from Aladdin Reagent Co., Ltd. (Shanghai, China). A deionized water Millipore filter was obtained from Millipore Q, USA (Millipore Q, Billerica, MA, USA).

### 2.2. Synthesis of NiS_2_ (MS) with Dual Sulfur Source

Cleaned carbon fiber paper (CFP), washed using deionized water and absolute ethanol, was prepared. Then, 1.2 mM of Ni(NO_3_)_2_·6H_2_O and 1.6 mM of MPS were mixed into the Teflon-lined autoclave with 25 mL deionized water and stirred for 15 min. After the above step, 0.8 mM of sulfur powder was added to the solution and stirred for 15 min slowly. After finishing this step, the CFP was placed into a container and was heated to 180 °C for 8 h. After this, the CFP was taken out and washed.

### 2.3. Synthesis of NiS_2_ with MPS

Cleaned carbon fiber paper (CFP), washed by deionized water and absolute ethanol, was prepared. Then, 1.2 mM of Ni(NO_3_)_2_·6H_2_O and 1.6 mM of MPS were added to the Teflon-lined autoclave with 25 mL deionized water and stirred for 15 min. After finishing this step, the CFP was placed into a container and heated to 180 °C for 8 h. After this, the CFP was taken out and washed.

### 2.4. Synthesis of NiS_2_ with S

Cleaned carbon fiber paper (CFP), washed by deionized water and absolute ethanol, was prepared. Then, 1.2 mM of Ni(NO_3_)_2_·6H_2_O was added to the Teflon-lined autoclave with 25 mL deionized water and stirred for 15 min. After the above step, 0.8 mM of sulfur powder was added to the solution and stirred for 15 min slowly. After finishing this step, put CFP was placed into a container and heated to 180 °C for 8 h. Finally, the CFP was taken out and washed.

### 2.5. Materials Characterization

X-ray diffractometer (XRD) by Bruker D8 Advance X-ray diffractometer (XRD) with Cu-Kα radiation (15° to 75°, 0.1° s^−1^) (Bruker Daltonics Inc., Karlsruhe, Germany). Raman measurements by Horiba LabRAM system (HORIBA, Ltd., Kyoto, Japan). SEM images by Scanning electron microscopy (FE-SEM; JSM-7000F JEOL Ltd., Tokyo, Japan). TEM and HRTEM images by JEOL type JEM2100 instrument (JEOL Ltd., Tokyo, Japan). XPS was performed on as-synthesized NiS_2_ by PHI5000 Versaprobe (Ulvac-Phi Inc., Kanagawa, Japan).

### 2.6. Electrochemical Measurements

A CHI760E electrochemical analyzer (CH Instruments, Chenhua Co., Shanghai, China) was used to analyze the performance of samples in 0.5 M H_2_SO_4_. A three-electrode system was used to test samples. The measured potentials were converted to a reversible hydrogen electrode (E(RHE) = E Hg/Hg_2_Cl_2_ + 0.241 + 0.0591 pH). Prior to testing, nitrogen had to be bubbled into H_2_SO_4_ solution to remove oxygen from the solution. The range of LSV test was from −0.8 to 0 V with the scan rate of 2 mV s^−1^. Tafel data could be calculated from the LSV test. Different scan rates of the CV test (5 to 90 mV s^−1^) were carried out to reveal the ECSA of samples. EIS measurements were carried out in a frequency range from 10^5^ to 0.1 Hz with an AC voltage of 5 mV.

## 3. Results and Discussion

**Figure 1:**
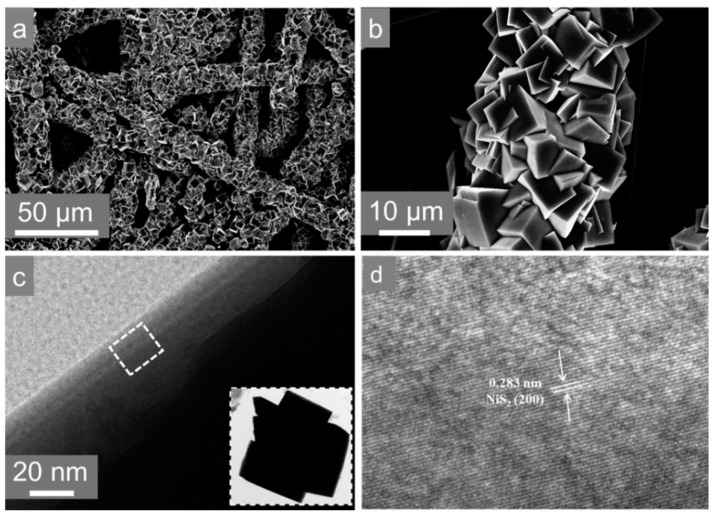
(**a**,**b**) Scanning electron microscopy images of NiS_2_; (**c**) TEM image and (**d**) HRTEM image of NiS_2_.

Figure 1a,b present scanning electron microscopy (SEM) images of NiS_2_ (MS) prepared by sulfur powder and MPS, forming a regular polyhedral block structure on the carbon fiber which was from the carbon fiber paper (CFP). Transmission electron microscopy image (TEM) captured from the edge of the sample is shown on Figure 1c. Figure 1d represents the HRTEM image of NiS_2_ (MS), with regular crystal plane spacing of 0.283 nm, corresponding to the (200) plane according to the following XRD card (JCPDS#65-3325) [24,25]. In order to compare the proposed NiS_2_ (MS) synthesis method, i.e., from dual sulfur sources, with other techniques, we also studied the morphology (Figures S1 and S2) of the synthesized material by two other techniques. As can be seen from Figure S1a,b, the carbon fiber surface also has some regularly shaped products stacked together. However, only a small amount of product adhered to the surface compared with the material synthesized by the dual sulfur source, which had a certain degree of negative influence on the conductivity between the substances. Figure S2 is SEM patterns of NiS_2_ synthesized by sulfur powder as the sole sulfur source. The resulting product was relatively small and scattered on the surface of the carbon fiber, which was in sharp contrast to the products synthesized by the previous two schemes. During the HER process, the other two samples easily detached from the carbon fiber, resulting in poor performance and stability. In comparison, the samples prepared by the dual sulfur source interlaced with each other provided a larger coverage of the electrode, and thus, improved resistance and catalytic performance.

**Figure 2:**
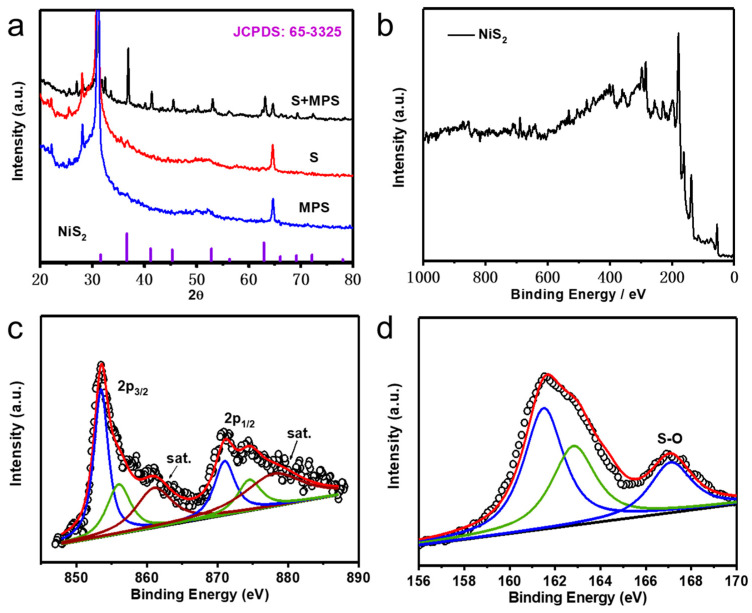
(**a**) XRD pattern of NiS_2_ prepared from three different sulfur sources; (**b**) X-ray photoelectron spectroscopy (XPS) survey spectra of NiS_2_; High-resolution XPS spectra of (**c**) Ni 2p, (**d**) S 2p of samples.

X-ray diffraction (XRD) analysis was performed in order to investigate the structure and composition of the obtained NiS_2_ [25]. Analyzing the shape of the peaks in the XRD picture, it can be seen that the obtained substance had good crystallinity. The diffraction peaks of 31.7°, 36.7°, 41.3°, 45.4°, 52.,9°, 56.4° and 62.9° corresponded to NiS_2_ planes according to JCPDS#65-3325 of (111), (200), (210), (211), (220), (221) and (311), respectively. The diffraction peak at 27° was attributed to the presence of carbon fiber [24]. The peak at 65° may be have been contributed to the presence of sulfur. Figure 2b represents the full x-ray photoelectron spectroscopy (XPS) spectra of NiS_2_ (MS) used for confirmation of its elemental composition [17,26]. As shown in Figure 2c, two spin-orbit doublets and two satellites can be deconvoluted from the Ni 2p spectrum, namely 853.5 eV and 856.1 eV corresponded to 2p3/2 of Ni^2+^ and Ni^3+^, and 871.1 eV and 874.5 eV were attributed to 2p1/2 of Ni^2+^ and Ni^3+^ respectively [17,26,27,28,29]. Figure 2d shows the deconvolution of S 2p peaks: the peak at 161.52 eV belonged to S 2p3/2, while that at 162.87 eV corresponded to S 2p1/2 of Ni-S bondings [30,31]. Due to the surface oxidation of NiS_2_, the peak at 166.7 eV could be attributed to S-O bonding [19].

**Figure 3:**
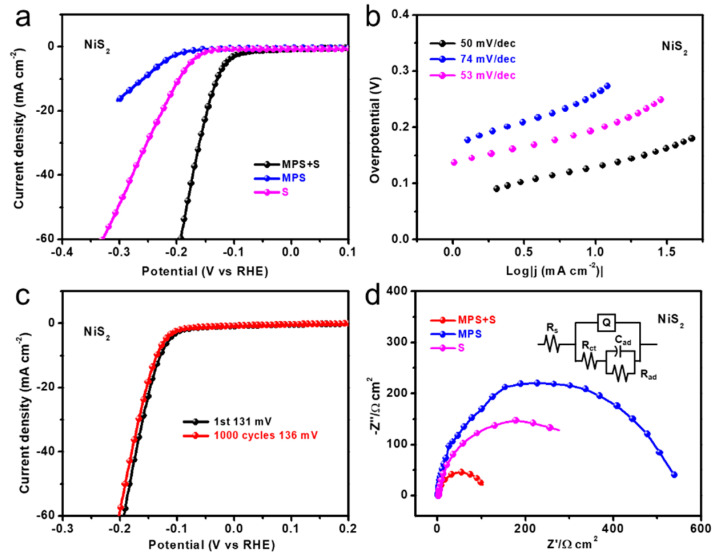
(**a**) LSV curves of NiS_2_ fabricated from different sulfur sources. (**b**) The corresponding Tafel plots of samples; (**c**) LSV curves before and after 1000 CV cycles and (**d**) electrochemical impedance spectroscopy of samples.

In order to further prove the applicability of the synthesized NiS_2_, electrochemical analysis was carried out to investigate the HER performance of the electrocatalyst in 0.5 M H_2_SO_4_ aqueous solution. Linear scan voltammetry (LSV) curves were measured to analyze the HER activity of NiS_2_, as shown in Figure 3a. The electrode based on NiS_2_ and grown on carbon paper from MPS and sulfur powder showed excellent HER performance, with an overpotential of 131 mV to drive −10 mA cm^−1^. Compared with the performance of the NiS_2_ synthesized using MPS or sulfur powder as the sulfur source, the overpotential was 197 mV and 261 mV, respectively. There may be two reasons for switching on the voltage gap. First, the increase in the loading of the substrate was obvious; provided more active sites for hydrogen adsorption and desorption. From the SEM image, it can be seen that there were many substrates on the surface that is synthesized by the dual sulfur source, which may have caused more defects in the boundary crystal planes, thereby improving the catalytic performance to a certain extent. It can be seen from the XRD pattern that the crystallinity of the sample prepared using the dual sulfur source was better. Combined with the TEM image of the sample, the high crystal plane (200) of the sample can be seen. This sample showed excellent performance in the process of electrocatalytic water decomposition. We summarized the overpotential of the HER of similar samples that have been published so far; Figure S3. Figure 3b shows the Tafel slope of 50 mV dec^−1^ for NiS_2_ with MPS and sulfur powder and the other two Tafel slopes of 53 mV dec^−1^ and 74 mV dec^−1^ for NiS_2_ with MPS or sulfur powder, respectively. The lowest overpotential and Tafel slope for NiS_2_ fabricated by MPS and sulfur powder indicated the best performance of hydrogen evolution reaction and high intrinsic catalytic activity supported by its superior exchange current density. To assess the stability of the catalytic performance of NiS_2_, cycling voltammetry (CV) was conducted. Thus, after 1000 cycles of stability testing at −10 mA cm^−1^, the performance loss was only 5 mV.

**Figure 4:**
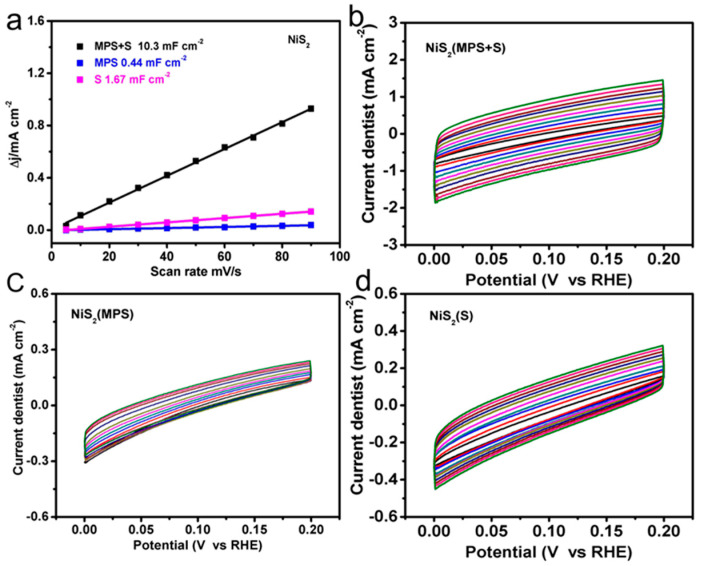
(**a**) The double-layer capacitance (C*_dl_*) calculated by liner fitting of the capacitive currents of different catalysts versus scan rate. (**b**–**d**) Cyclic voltammograms of NiS_2_ prepared by different experimental schemes.

Electrochemical impendence spectroscopy (EIS) was used to assess the charge transfer performance of the synthesized NiS_2_, which also determined the double layer capacitance [1,16]. The Nyquist plots (Figure 3d) confirmed that the NiS_2_ fabricated from MPS and sulfur powder showed excellently smaller charge transfer resistance (R*_ct_*), compared to other two materials, in the experiment and simulation in 0.5 M H_2_SO_4_ solution. The experimental data was fitted according to the electrical model presented in the inset of Figure 3d. C*_ad_* and R*_ad_* represent adsorption capacitance and adsorption resistance, respectively. The lowest charge transfer resistance of 53 Ω for NiS_2_ prepared by MPS and sulfur powder was observed from the fitting experiment data in Figure 3d. The charge transfer resistances of NiS_2_ with only MPS and only sulfur powder were 178 Ω and 258 Ω, respectively. Contrasting the value of R*_ct_* of the samples, it can be observed that the NiS_2_ fabricated by MPS and sulfur powder had improved electrical conductivity, allowing electrochemical processes to occur on the surface. Nonfaradaic double-layer capacitance (C*_dl_*) was calculated for further analysis of the NiS_2_ activity. The C*_dl_* of NiS_2_ fabricated by the dual sulfur source was 10.3 mF cm^−2^, and the C*_dl_* of the material prepared by MPS and sulfur powder were 0.44 mF cm^−2^ and 1.67 mF cm^−2^, respectively. Different scan rates of cyclic voltammetry measurement were carried out in order to determine the electrochemical surface area (ECSA) of NiS_2_, as shown in Figure 4a. The catalytic performance of the working electrode was normalized to 1 cm^2^. Figure 4a–d show cyclic voltammograms of NiS_2_ prepared by different experimental schemes which were measured in the nonfaradaic capacitance current range with different scan rates.

## 4. Conclusions

In this work, we proposed a one-step hydrothermal method for NiS_2_ (MS) synthesis from dual sulfur sources and applied the obtained material as a catalyst for HER. The resulting material had a regular morphology with more specific surface area and active sites. The electrochemical performance and stability of NiS_2_ prepared by the hydrothermal method improved significantly. We believe that our findings will have a positive impact on the industrial application of nonprecious metal catalysts.

## Supplementary Materials

The following are available online at https://www.mdpi.com/2079-4991/10/11/2115/s1, Figure S1. (a) and (b) are SEM images of NiS_2_ fabricated by MPS as the only sulfur source. Figure S2. (a) and (b) are SEM patterns of the product which prepared by sulfur powder only. Figure S3. Contrast of HER activity of electrocatalysts of similar materials.
