# Supplementary material for: Hydrothermal Synthesis of Polyhedral Nickel Sulfide by Dual Sulfur Source for Highly-Efficient Hydrogen Evolution Catalysis
Source: Nanomaterials (Basel). 2020 Oct 24;10(11):2115. doi: 10.3390/nano10112115 (PMC7692512; doi:10.3390/nano10112115)
Supplement: Supplementary file 1 [file nanomaterials-10-02115-s001.pdf]

nanomaterials

Supplementary Information

Hydrothermal synthesis of polyhedral nickel sulfide by dual sulfur source for high-efficient hydrogen evolution catalysis

Yuan Gao [1], Ka Wang [1], Zixia Lin [2], Haizeng Song [3], Xiaomeng Duan [4], Zehui Peng [1] and Shancheng Yan [1,*]

[1] School of Geography and Biological Information, Nanjing University of Posts and Telecommunications, Nanjing 210023, China; 1218063835@njupt.edu.cn (Y.G.); 1217063729@njupt.edu.cn (K.W.); 1019172222@njupt.edu.cn (Z.P.)
[2] Testing center, Yangzhou University, Yangzhou 225009, China; zxlin@yzu.edu.cn
[3] School of Electronic Science and Engineering, Nanjing University, Nanjing 210093, China; dg1823028@smail.nju.edu.cn
[4] Jiangsu National Synergetic Innovation Center for Advanced Materials (SICAM), Institute of Advanced Materials (IAM), Nanjing University of Posts & Telecommunications, Nanjing 210023, China; 1218063822@njupt.edu.cn
* Correspondence: yansc@njupt.edu.cn

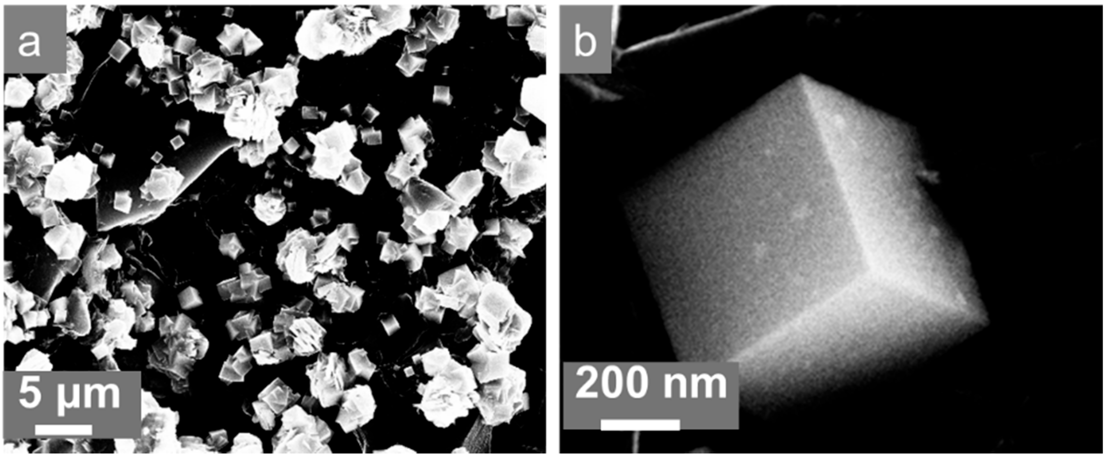

Figure S1. (**a**) and (**b**) are SEM images of NiS_2 fabricated by MPS as the only sulfur source.

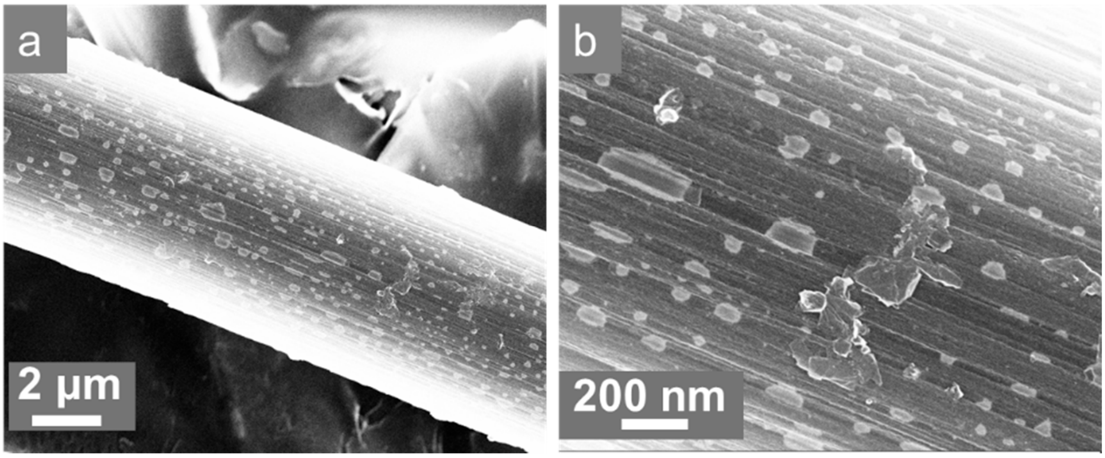

Figure S2. (**a**) and (**b**) are SEM patterns of the product which prepared by sulfur powder only.

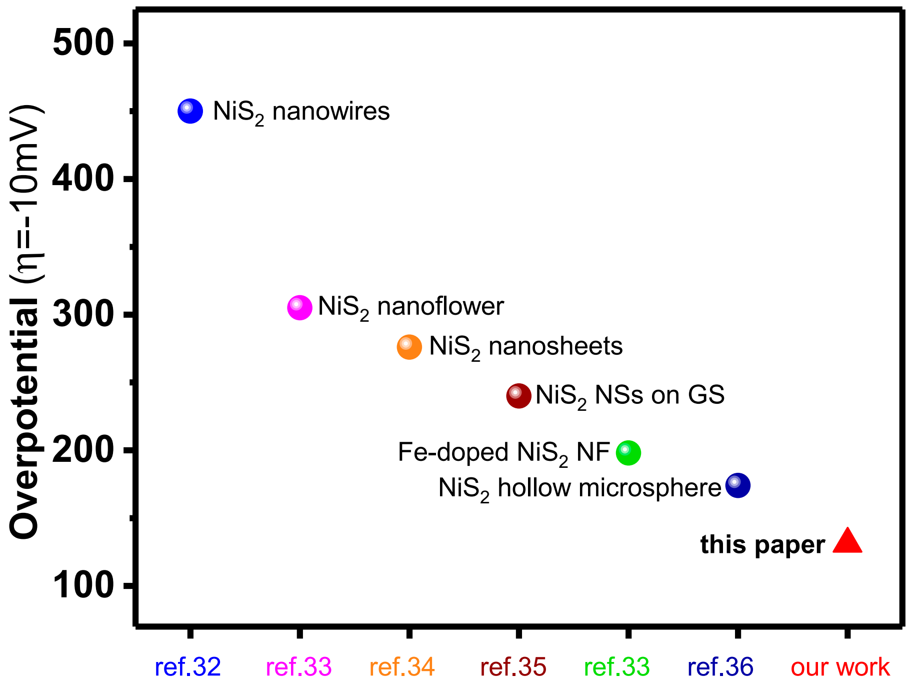

Figure S3. Contrast of HER activity of electrocatalysts of similar materials.
